# Supplementary material for: Building capacity of primary health care workers and clients on COVID-19: Results from a web-based training
Source: PLoS One. 2022 Oct 7;17(10):e0274750. doi: 10.1371/journal.pone.0274750 (PMC9543989; doi:10.1371/journal.pone.0274750)
Supplement: Attachment — Submitted filename: PONE-D-22-16429_reviewer.pdf [file pone.0274750.s002.pdf]

PLOS ONE

The Editor-in-Chief PLoS One
FULL TITLE
Building capacity of Primary Health Care workers and Clients on COVID-19: Results from a web-based training

--Manuscript Draft--

**Manuscript Number:**	PONE-D-22-16429
**Article Type:**	Research Article
**Full Title:**	The Editor-in-Chief PLoS One<br>FULL TITLE<br>Building capacity of Primary Health Care workers and Clients on COVID-19: Results from a web-based training
**Short Title:**	The effect of virtual training on the knowledge of health workers on COVID-19
**Corresponding Author:**	Olumuyiwa O Odusanya, MBBS, MPH, FMCPH<br>Lagos State University College of Medicine<br>Lagos, Lagos NIGERIA
**Keywords:**	Capacity-building, COVID-19, behaviour change, knowledge, attitudes, preventive practices
**Abstract:**	Background. Health care workers (HCWs) in the first line of care play critical roles in providing the correct information about the coronavirus disease to the community. The objective of the study was to determine the effect of virtual training on the knowledge, attitude, and practices among PHC workers and their clients in the prevention and control of coronavirus disease.<br>Methods. A quasi-experimental intervention virtual training, using a before and after design amongst HCWs and clients was conducted at primary health care facilities in two Local Government Areas of Lagos State. The study instruments were pre-questionnaires for both HCWs and their clients. which investigated knowledge of symptoms, modes of disease transmission, methods of prevention, and preventive practices. Changes in knowledge, attitudes, and practices were compared pre-and post-intervention. The level of significance was set at $p < 0.05$<br>Results. Sixty-six HCWs (out of 100 recruited at baseline) and 133 clients (out of the initial 226) completed the study. The mean ages of the HCWs and clients were 39.2±9.9 and 30.9±5.0 respectively. At the baseline, the HCW's knowledge was good in the domains of symptoms, modes of transmission, and preventive measures. The training led to a higher but not significant ($p> 0.05$) increase in the level of knowledge. Contact with trained HCWs was found to lead to significantly ($P < 0.001$) higher levels of knowledge, attitudes, and preventive practices. amongst clients.<br>Conclusion. The training was effective in improving the knowledge of both the trained HCWs and their clients.
**Order of Authors:**	Olumuyiwa O Odusanya, MBBS, MPH, FMCPH
	Adeyinka Adeniran
	Omowunmi Bakare
	Babatunde Odugbemi, FMCPH
	Oluwatoyin Enikuomehin, PhD
	Olugbenja Jeje, MBBS
	Angela Emechebe, MBBS
**Additional Information:**	
**Question**	**Response**
**Financial Disclosure**	OOO received a research grant from Lagos State Research and Innovation Council. website. https://lasric.lagosstate.gov.ng

Powered by Editorial Manager® and ProduXion Manager® from Aries Systems Corporation

Enter a financial disclosure statement that describes the sources of funding for the work included in this submission. Review the submission guidelines for detailed requirements. View published research articles from *PLOS ONE* for specific examples.<br><br>This statement is required for submission and **will appear in the published article** if the submission is accepted. Please make sure it is accurate.<br><br>**Unfunded studies**<br>Enter: *The author(s) received no specific funding for this work.*<br><br>**Funded studies**<br>Enter a statement with the following details:<br>• Initials of the authors who received each award<br>• Grant numbers awarded to each author<br>• The full name of each funder<br>• URL of each funder website<br>• Did the sponsors or funders play any role in the study design, data collection and analysis, decision to publish, or preparation of the manuscript?<br>• **NO** - Include this sentence at the end of your statement: *The funders had no role in study design, data collection and analysis, decision to publish, or preparation of the manuscript.*<br>• **YES** - Specify the role(s) played.<br><br>* typeset	The funders had no role in study design, data collection and analysis, decision to publish, or presentation of the manuscript
**Competing Interests**<br><br>Use the instructions below to enter a competing interest statement for this submission. On behalf of all authors, disclose any competing interests that could be perceived to bias this work—acknowledging all financial support and any other relevant financial or non-financial competing interests.<br><br>This statement is required for submission and **will appear in the published article** if the submission is accepted. Please make	The authors have declared that no competing interests exist

sure it is accurate and that any funding sources listed in your Funding Information later in the submission form are also declared in your Financial Disclosure statement.<br><br>View published research articles from *PLOS ONE* for specific examples.<br><br>**NO authors have competing interests**<br><br>Enter: *The authors have declared that no competing interests exist.*<br><br>**Authors with competing interests**<br><br>Enter competing interest details beginning with this statement:<br><br>*I have read the journal's policy and the authors of this manuscript have the following competing interests: [insert competing interests here]*<br><br>* typeset	
**Ethics Statement**<br><br>Enter an ethics statement for this submission. This statement is required if the study involved:<br><br>• Human participants<br>• Human specimens or tissue<br>• Vertebrate animals or cephalopods<br>• Vertebrate embryos or tissues<br>• Field research<br><br>Write "N/A" if the submission does not require an ethics statement.<br><br>General guidance is provided below. Consult the submission guidelines for detailed instructions. **Make sure that all information entered here is included in the Methods section of the manuscript.**	Ethics approval was obtained from the Health and Research Ethics Committte of the Lagos State Univeristy Teaching Hospital

**Format for specific study types**<br><br>**Human Subject Research (involving human participants and/or tissue)**<br>• Give the name of the institutional review board or ethics committee that approved the study<br>• Include the approval number and/or a statement indicating approval of this research<br>• Indicate the form of consent obtained (written/oral) or the reason that consent was not obtained (e.g. the data were analyzed anonymously)<br><br>**Animal Research (involving vertebrate animals, embryos or tissues)**<br>• Provide the name of the Institutional Animal Care and Use Committee (IACUC) or other relevant ethics board that reviewed the study protocol, and indicate whether they approved this research or granted a formal waiver of ethical approval<br>• Include an approval number if one was obtained<br>• If the study involved *non-human primates*, add *additional details* about animal welfare and steps taken to ameliorate suffering<br>• If anesthesia, euthanasia, or any kind of animal sacrifice is part of the study, include briefly which substances and/or methods were applied<br><br>**Field Research**<br><br>Include the following details if this study involves the collection of plant, animal, or other materials from a natural setting:<br>• Field permit number<br>• Name of the institution or relevant body that granted permission	
**Data Availability**<br><br>Authors are required to make all data underlying the findings described fully available, without restriction, and from the time of publication. PLOS allows rare exceptions to address legal and ethical concerns. See the PLOS Data Policy and FAQ for detailed information.	Yes - all data are fully available without restriction

A Data Availability Statement describing where the data can be found is required at submission. Your answers to this question constitute the Data Availability Statement and **will be published in the article**, if accepted.<br><br>**Important:** Stating 'data available on request from the author' is not sufficient. If your data are only available upon request, select 'No' for the first question and explain your exceptional situation in the text box.<br><br>Do the authors confirm that all data underlying the findings described in their manuscript are fully available without restriction?	
**Describe where the data may be found in full sentences. If you are copying our sample text, replace any instances of XXX with the appropriate details.**<br><br>• If the data are **held or will be held in a public repository**, include URLs, accession numbers or DOIs. If this information will only be available after acceptance, indicate this by ticking the box below. For example: *All XXX files are available from the XXX database (accession number(s) XXX, XXX.).*<br>• If the data are all contained **within the manuscript and/or Supporting Information files**, enter the following: *All relevant data are within the manuscript and its Supporting Information files.*<br>• If neither of these applies but you are able to provide **details of access elsewhere**, with or without limitations, please do so. For example:<br><br>*Data cannot be shared publicly because of [XXX]. Data are available from the XXX Institutional Data Access / Ethics Committee (contact via XXX) for researchers who meet the criteria for access to confidential data.*<br><br>*The data underlying the results presented in the study are available from (include the name of the third party*	data set is uploaded

and contact information or URL).<br>• This text is appropriate if the data are owned by a third party and authors do not have permission to share the data.<br><br>* typeset	
Additional data availability information:	

Powered by Editorial Manager® and ProduXion Manager® from Aries Systems Corporation

Manuscript

Click here to access/download;Manuscript;odusanya_eta-_plosone.docx

Title

Building capacity of Primary Health Care workers and Clients on COVID-19: Results from a web-based training

Authors

Olumuyiwa. O. Odusanya, *[1] Adeyinka Adeniran, [1] Omowunmi Q. Bakare,[1] A. Babatunde A. Odugbemi, [1] Oluwatoyin Enikuomehin, [2] Olugbenga O. Jeje, [3] Angela Emechebe.[3]

Address.

1. **Department of Community Health and Primary Health Care, Lagos State University College of Medicine, Ikeja**

2. **Department of Computer Science, Lagos State University, Ojo**

3. **Department of Community Health and Primary Health Care, Lagos State University College Teaching Hospital, Ikeja.**

***Corresponding Author**
Email. profodusanya@gmail.com

Short title. The effect of virtual training on the knowledge of health workers on COVID-19

Abstract

Background. Health care workers (HCWs) in the first line of care play critical roles in providing the correct information about the coronavirus disease to the community. The objective of the study was to determine the effect of virtual training on the knowledge, attitude, and practices among PHC workers and their clients in the prevention and control of coronavirus disease.

Methods. A quasi-experimental intervention virtual training, using a before and after design amongst HCWs and clients was conducted at primary health care facilities in two Local Government Areas of Lagos State. The study instruments were pre-questionnaires for both HCWs and their clients. which investigated knowledge of symptoms, modes of disease transmission, methods of prevention, and preventive practices. Changes in knowledge, attitudes, and practices were compared pre-and post-intervention. The level of significance was set at $p < 0.05$

Results. Sixty-six HCWs (out of 100 recruited at baseline) and 133 clients (out of the initial 226) completed the study. The mean ages of the HCWs and clients were 39.2±9.9 and 30.9±5.0 respectively. At the baseline, the HCW's knowledge was good in the domains of symptoms, modes of transmission, and preventive measures. The training led to a higher but not significant ($p> 0.05$) increase in the level of knowledge. Contact with trained HCWs was found to lead to significantly ($P < 0.001$) higher levels of knowledge, attitudes, and preventive practices. amongst clients.

Conclusion. The training was effective in improving the knowledge of both the trained HCWs and their clients.

Keywords. Capacity-building, COVID-19, behaviour change, knowledge, attitudes, preventive practices

Introduction

The current pandemic caused by novel Coronavirus (2019-nCoV), known as coronavirus disease (COVID-19) was first reported in December 2019, as a cluster of acute respiratory illnesses in Wuhan, Hubei Province, China, from where it spread rapidly to over 198 countries.[1] As of May 30, 2022, the number of confirmed cases reported globally to the World Health Organization was 526,182, 662 confirmed cases, and 6,286,057 deaths[2] and in Nigeria, the number of confirmed cases is 256,028 with 3,143 deaths. [2] Effecting behaviour change about COVID-19 is partly dependent upon the level of knowledge possessed by health care workers (HCWs) and the community. Various studies have been conducted to determine the level of knowledge in both groups.

In the United Arab Emirates (UAE), a study amongst 453 HCWs showed that 61% had a poor level of knowledge of the means of transmission, 64% had poor knowledge of symptoms and 75% had positive perceptions about the disease. Age and professional cadre were associated with inadequate knowledge and poor perception.[3] In Yemen, a study amongst 514 HCWs found that physicians had statistically significantly higher levels of knowledge than other professional groups ($p < 0.001$). HCWs in intensive care units and emergency departments were better prepared than in other areas of care probably because of continuing exposure and training about the disease.[4]

A group of workers from Mozambique reported knowledge of COVID-19 amongst HCWs. They found that a quarter correctly identified all three common symptoms (shortness of breath, dry persistent cough, and fever), 2% correctly identified groups at risk, 16% knew three means of transmission and 39% knew three methods of prevention. The response rate in the study was 28%.[5] In Uganda, 69% of HCWs had sufficient knowledge, and 74% reported practising good preventive measures.[6] In Ethiopia, a facility-based cross-sectional study amongst 379 HCWs using multi-stage sampling techniques reported that 75% had adequate knowledge, 84% had positive attitudes and 69% had good preventive practices. Factors associated with good knowledge were higher levels of education (a master’s degree), training on COVID-19, and working in a comprehensive specialist hospital. Good preventive practices in the study were associated with the source of information, working in primary hospitals, and possessing good knowledge. Work experience and knowledge were significantly associated with attitudes.[7]

Nine out of ten HCWs in a teaching hospital in Bayelsa State, Southern Nigeria had good knowledge, 90% had good hand hygiene practices but 52% used face masks.[8] This is not

unexpected due to the concentration of highly skilled and knowledgeable HCWs in teaching hospitals. The proportion of HCWs with good knowledge in a study from Northern Nigeria was 83%. Eight out of ten had positive attitudes and 88% had good preventive practices. The odds of good knowledge were significantly lower among community health officers compared with doctors. Positive attitudes were predicted by good knowledge, older age (fifth decade), higher education, and Christian faith. A significant association was also found between good preventive practices and good knowledge. [9]

A study amongst 589 participants recruited using snowball sampling in North-Central Nigeria reported that 99.5% had good knowledge of COVID-19. The source of information was through social media (56%) and television (28%). Almost 80% had positive and 82% used face masks. A significant relationship was found between knowledge of COVID-19 and attitudes towards preventive practices. A limitation of the study was that only literate subjects and those with internet access could participate in addition to the use of non-probability sampling methods.[10]

A study amongst 961 young Nigerians showed that 92% had good knowledge about clinical symptoms of COVID such as fever, fatigue, dry cough, and body pains but only 31% reported wearing face masks. A strong association was found between knowledge and age groups. A higher level of education was found to be significantly associated with the use of preventive measures such as wearing face masks and regular hand washing.[11] In Kano, Northern Nigeria, 30% of respondents had good knowledge, 11% had good attitudes and 26% had good preventive practices, which are lower than values in other parts of Nigeria. [12]

A cross-sectional study amongst mothers of under-five children attending a teaching hospital in Enugu showed that 65% were aware that asymptomatic persons could transmit COVID-19, 72% thought children under five years could contract the infection and 43% had good perceptions about COVID-19.[13] Eighty-six percent of pregnant women (n=527) in Northern Ghana were found to have adequate knowledge about COVID-19 and 49% had positive preventive practices. Factors associated with adequate knowledge were having at least a primary six education, living in an urban area, and having received health education about the disease at a health facility. Having good preventive practices was associated with having at least a primary six education, living in an urban area, and pregnant women with a chronic disease while multiparty was negatively associated with it.[14]

With varying levels and gaps in the knowledge and practices of HCWs and the public, it becomes important to build the capacity of both groups if COVD-19 is to be effectively controlled. HCWs at the first level of care attend to the larger segments of the population and are in the best position to influence them. Positioning HCWs to be able to assume this role requires continuous training. Formal means of training such as face-to-face contact lectures may not be suitable during the pandemic. Thus, a virtual training approach becomes useful. Moreover, there has been very limited training for HCWs, especially at the first level of care.[15] Therefore, the goal of the study was to determine the knowledge, attitudes, and preventive practices of HCWs, train them and assess the effect of the training on their clients' knowledge, attitudes, and preventive practices towards the infection.

Materials and Methods

Settings

Lagos State is one of the 36 states in Nigeria and remains the economic nerve centre of the nation. Politically, it has 20 Local Government Areas (LGAs), 37 Local Council Development Areas (LCDAs) and 376 Wards. The population is estimated to be over 20 million. The health care of its residents is overseen by the Lagos State Ministry of Health. The Primary Health Care (PHC) level of care is managed by the Lagos State Primary Health Care Board.

The study was conducted in Ikeja and Alimosho local government areas (LGAs) of Lagos State. Ikeja LGA is the capital of Lagos State. The LGA is bound in the north by Ifako-Ijaiye LGA, the south by Oshodi-Isolo LGA, the east by Agege LGA, and the west by Kosofe LGA. The LGA has an estimated population of 460,000 residents. There is a mixture of public and private health facilities in the LGA. Government-owned health facilities include 18 PHC centres and the Lagos State University Teaching Hospital (LASUTH). Alimosho LGA is the largest and has an estimated population of 1,469,134 residents. Health services are also delivered here through a mix of government-owned and private health facilities. The LGA hosts a government-owned secondary health facility, Alimosho General Hospital, Igando, and 33 PHCs.

Trial design

A before and after quasi-experimental study approach was used to conduct the study amongst HCWs and their clients (mothers of under-five children). The study had three phases: baseline, intervention, and post-intervention. The intervention phase was designed to meet identified gaps from the baseline phase.

Sample size

The sample size was calculated using the appropriate formula for sample size calculation for the intervention study.[16] A minimum of 100 health care workers (50 in each LGA) and 200 community members (100 in each LGA) were required to achieve the study objectives.

Participants eligibility criteria

All health care workers above 18 years involved in patient care who were willing to participate in all phases of the study but PHC workers not willing to give consent were excluded. The

clients were mothers of under-five children utilizing the PHCs who were willing to participate in all phases of the study.

Sampling techniques

A multi-stage sampling method was used to recruit participants for this study. A simple random sampling method was used to select two (2) out of 20 LGAs in the state. The selected LGAs were Ikeja and Alimosho. PHCs were selected based on having a high patient load and all eligible health workers in the selected facilities were recruited into the study. Eligible clients were recruited consecutively.

Survey instruments

Two almost identical study instruments were used to obtain data. The first was a PHC workers questionnaire and the other was a client questionnaire. Both were designed to elicit the knowledge, attitudes, and preventive practices of COVID-19 among the respondents. The questionnaires were developed based on current literature on the subject.[17-18]

The questionnaires consisted of three parts. The first part collected information on the biodata and work experience of the health workers including whether they had attended courses on COVID-19 and infection prevention and control and their sources of information on COVID-19. The second part which consisted of 31 questions was further sub-divided into 3 parts consisting of "yes, No and don't know options on their knowledge of COVID-19 and Likert-scale questions on their attitudes and preventive practices toward COVID-19. The knowledge questions (n=11) were on the causative agent, incubation period, symptoms, mode of spread, and severity of COVID-19. Attitude questions (n=9) were responses to the likelihood if the participants had COID-19 and to others suspected of having the infection. The attitudes were assessed using a 5-point Likert Scale: "strongly disagree, disagree, neutral, agree, and strongly agree". Preventive practice questions (n=9) elicited information on what respondents did to prevent the disease such as hand washing, use of face masks, and physical distancing. The preventive practices domain was assessed using a 4-point Likert Scale: "always, often, sometimes and never". The third part consisting of 6 questions was on vaccine acceptability.

Face and content validity was done by the research team. The reliability coefficients of the HCWs tool sub-scales were: knowledge, $\alpha = 0.63$; attitude, $\alpha = 0.60$; and practice, $\alpha = 0.86$. The reliability coefficients for the clients' tool were: knowledge, $\alpha = 0.82$; attitude, $\alpha = 0.67$;

and practice, α = 0.80. Pre-testing of the study instruments was done amongst 20 HCWs in an LGA not involved in the study and who met the eligibility requirements of the study. This was done to test the flow of questions, and clarity of the questions to the interviewer.

Conduct of the study and intervention

The project was carried out in three phases: baseline (pre-intervention), intervention, and post-intervention phases. The baseline survey was to determine the level of COVID-19 knowledge, attitude, and practices among health workers using the pre-tested self-administered questionnaire. Questionnaires were administered to clients on their first visit to the PHC to assess their knowledge, attitudes, and preventive practices against COVID-19. The tool for the client survey was interviewer-administered and collected on the kobo collect form electronically.

The intervention was developed based on the theory of change model a concept that is used to explain how and why change occurs.[19] There are several approaches to the use of the theory. It is a process that focuses on the need, the assumptions, the desired interventions, and the changes desired. For the study, the problem was inadequate knowledge of COVID-19 amongst the HCWs and clients. The assumptions were willingness to learn and building upon the experience of the HCWs. The key constructs were root cause analysis to identify the gaps; input which is the web-based training programmes; the output was the increased knowledge amongst HCWs and clients and some measure of behaviour change. The outcome constructs, sustained behaviour change and reduced transmission of COVID-19 in the community would. occur beyond the duration of the study.

The intervention was a virtual training on COVID-19 for PHC workers. A website was developed for the training. The website was registered, and the name was approved by the regulatory authorities. The website address was www.healthworkerstraining.com.ng The training was conducted to update the knowledge of the PHC workers on COVID-19 and meet the gaps identified from the baseline survey. The website contained videos, presentation slides, quizzes, case scenarios, and problem-solving sessions with provisions for discussion and comments.

There were five main modules in addition to an introductory session. Each module lasted about 25 minutes. The modules were: epidemiology, risk communication, community engagement,

prevention of COVID-19, and vaccination against COVID-19. The delivery methods consisted of a mixture of lectures, practical demonstrations, assignments, and question, and answer sessions. Each module had pre-test and post-test multiple-choice questions (one in four best answer options) to monitor participants' progress. The training was self-paced and delivered entirely online.

Six research assistants with a minimum of Ordinary National Diploma (OND) qualification, fluent in both Yoruba and English languages were trained for two days on the aim and objectives of the study, sampling procedures, administration of the questionnaire, with practical sessions.

Outcomes

The outcomes were changes in the knowledge, attitudes, and preventive practices which were scored. For the knowledge domain, each correct answer was assigned one mark and a wrong answer zero. The maximum knowledge score of HCWs was 33. The attitude of the HCWs was scored, the range was from one to five, and the maximum score was 45. The preventive practices were scored, the range from one to four, and the maximum score for the preventive practice domain was 36. Categorization of knowledge, attitudes and preventive practices was done into good if the scores were at least 66%.[38] Scoring for the clients was like that of the HCWs except that the maximum knowledge score was 27 and the attitude score was 40.

Data Management

Data were entered and cleaned on Microsoft Excel and imported to STATA where it was analyzed using STATA SE Version 12. Changes in knowledge, attitudes and practices of the HCWs and the clients were compared pre-intervention and post-intervention using paired t-test (when the data were normally distributed) and Wilcoxon signed-rank test (when the data were not normally distributed). Associations between the socio-demographic characteristics of respondents and their post-intervention COVID-19 knowledge, attitude, and preventive practices were assessed using an independent sample t-test and one-way analysis of variance (ANOVA) when data were normally distributed or Wilcoxon signed-rank and Kruskal-Wallis equality-of-populations rank test as appropriate when the data were not normally distributed. The level of significance was set at $P < 0.05$.

Ethical considerations:

The project was conducted in compliance with Good Clinical Practice standards. Ethical approval was obtained from the Health Research Ethics Committee of the Lagos State University Teaching Hospital (REF NO: LREC/06/10/1501). Written informed consent was obtained from all the participants after explaining the study's aim and objectives.

Results.

PHC Workers survey

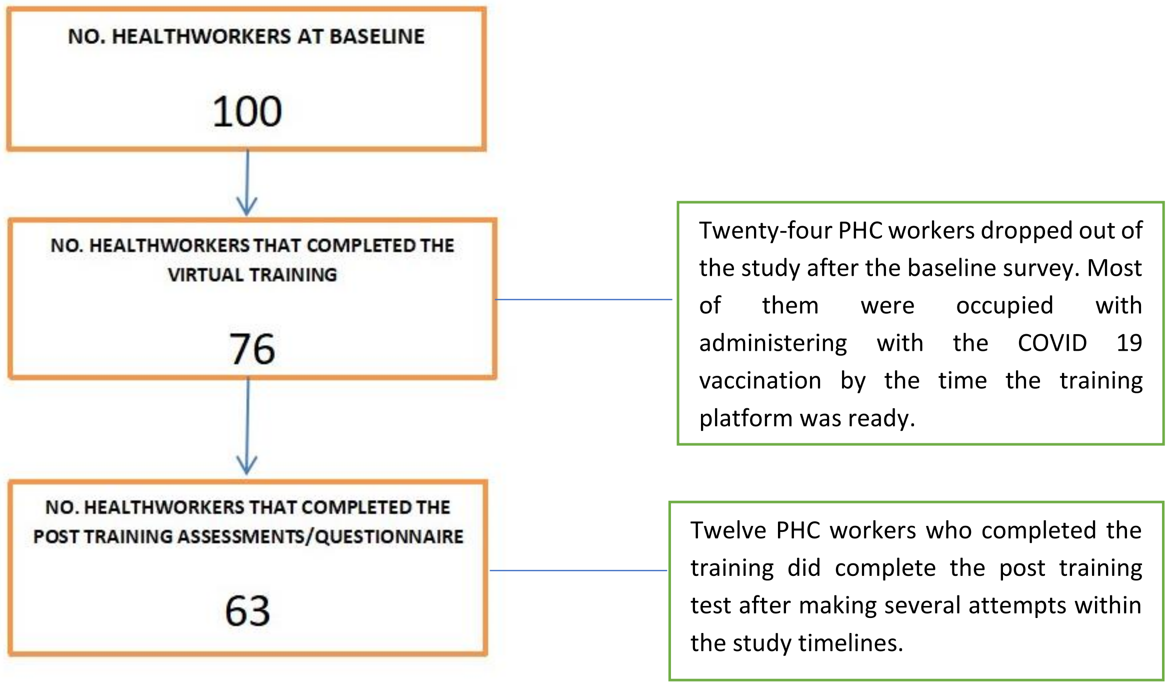

Figure 1. Study Flow Chart for PHC Workers

Sixty-three primary healthcare workers who completed the virtual training and post-training assessment were interviewed after and had post-training interaction with clients enrolled for this study out of the 100 that were enrolled at baseline giving a 63% completion rate (Figure 1).

The socio-demographic characteristics of the HCWs are shown in Table 1. The mean ages at the baseline and the end of the study were 40.8±9.6 and 39.2±9.9 years respectively. Females constituted 85% of the group. Half of the HCWS were nurses. The mean years of work experience at baseline and the end of the study were 12.8±9.4 and 12.5±10.0 years respectively. Table 2 displays information about awareness and knowledge of the HCWs on the disease pre- and post-training. About 95.2% had heard of the Covid-19 virus during the baseline study and all post-training. About 87% at baseline and 98.4% at post-training knew that the incubation

period was 1-14 days. The level of knowledge was high on symptoms, modes of transmission, and preventive measures. Sixty -two (98.4%) of the HCWs were willing to take the COVID-19 vaccine at baseline and all were willing at the end of the study.

There were no statistically significant differences in the mean COVID 19 knowledge, attitude, and preventive practices scores of PHC workers before and after training, even though they had higher scores (see Table 3). Table 4 shows that health workers more than 30 years had a significantly higher mean knowledge score (28.80± 1.71) than those who were 30 years or less (27.81±2.64). Marital status and LGA of health workers were statistically significantly associated with knowledge scores. The preventive practice scores of the HCWs were not significantly associated with any attributes of the participants as shown in Table 5.

Client's survey

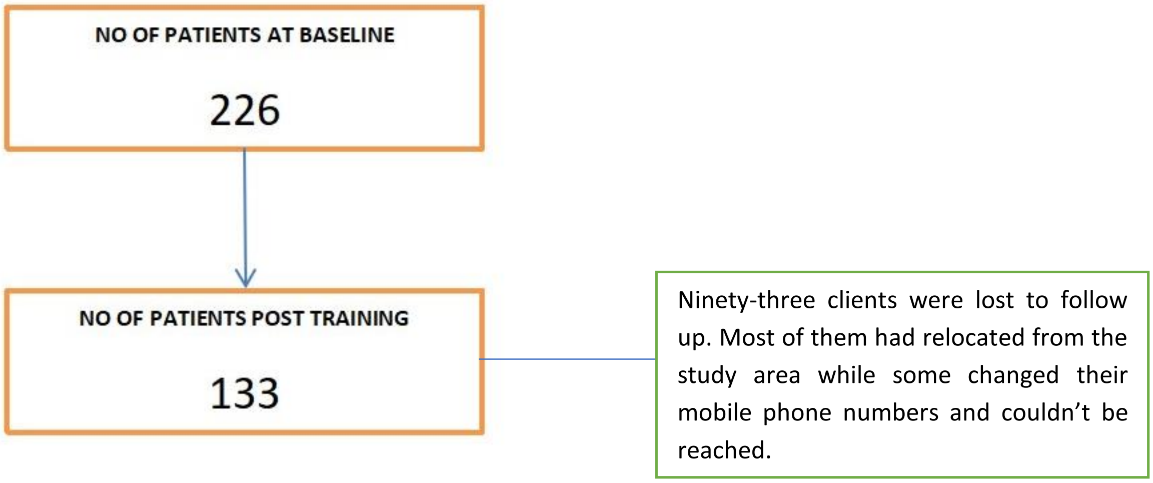

Figure 2. Study Flow Chart for clients

One hundred and thirty-three clients completed the study and were interviewed after their interactions with trained PHC workers out of the 226 that were surveyed at baseline, giving a completion rate of 58.9% (Figure 2).

At baseline, more than half (54.2%) of the clients were either 30years or less, with a mean age of 30.5 ± 5.8 years. The mean age at the end of the study was 30.9 ± 5.0 years. The majority (82%) were employed, most (78%) earned ₦50,000 or less and 50% had a secondary level of education (Table 6). The awareness and knowledge of the clients are shown in Table 7. At the baseline study, 98.5% of clients had heard of the Covid-19 virus and among those who have heard, 80.2% stated that the Covid-19 virus was highly infectious, and 73% knew that the virus can be transmitted by asymptomatic patients. The interaction with trained PHC workers led to an increase in all aspects of the knowledge enquired about: symptoms, transmission, and prevention. The willingness to accept COVID-19 vaccines amongst the clients increased from 60 (45.1%) to 92 (69.2%) after meeting with trained HCWs.

Table 8 shows that among those that there were significantly higher scores in the knowledge, attitude, and practices scores of clients after interaction with trained PHC workers ($p<0.001$). Marital status and PHC location were statistically significantly associated with clients' knowledge scores after meeting with trained PHC workers. ($p<0.05$). The single respondents had a better knowledge compared to the married ones. Clients attending PHCs in Ikeja LGA had significantly better knowledge compared with those attending PHCs in Alimosho LGA (see Table 9). Table 10 displays information about the association between preventive practice scores and clients' sociodemographic characteristics. Only LGA of the PHC was statistically significantly associated with the practice scores of respondents after consultation encounter with a trained primary health worker. Clients attending PHCs in Alimosho LGA had statistically significant better practices compared to those attending PHCs in Ikeja LGA.

Discussion

This intervention study set out to determine the knowledge of, attitudes to, and preventive practices of HCWs and their clients at baseline and to evaluate the effect of the training in both groups.

At baseline, HCWs' knowledge was high, attitudes were positive and preventive practices were good. This high level of knowledge agrees with high levels reported from the UAE,[3] Uganda,[6] Ethiopia,[7] and Nigeria.[8,9] It is higher than the levels reported from Yemen[4] and Mozambique.[5] Nine out of ten HCWs correctly identified the common symptoms such as cough, shortness of breath and cough. The high level of knowledge is probably a reflection of the multiple sources of information and training received by the HCWs since the pandemic began. At the time of the study, the pandemic had lasted for over a year in Nigeria, thus information and awareness on COVID were abundant. Therefore, it was not surprising that HCWs had a high level of knowledge. Had the study been conducted very early during the pandemic, perhaps the level of knowledge may not have been as high. This high level of knowledge gives assurance that the health workers were more likely to give correct information to their clients and help further in changing their behaviour. Nevertheless, there were some gaps in the knowledge of the epidemiology of the disease amongst the HCWs which formed the basis for the intervention

The clients' levels of knowledge at baseline were fair to moderate. Common symptoms of the disease such as fever, cough and shortness of breath were known although knowledge of more specific ones like anosmia and ageusia was lower. The level of knowledge of the clients is comparable with studies in Pakistan,[20] but much higher than was reported from Kano, northern Nigeria[12] which may be due to lower access to information and lower levels of literacy in Kano. The proportion of clients in this study who were aware that an asymptomatic person could transmit COVID-19 was similar to a study amongst mothers of under-five children in Enugu.[13] In addition, the 70% rate of good preventive practices found at baseline was much higher than the 49% reported amongst pregnant women in Northern Ghana.[14] The vaccine uptake was low probably because of the non-availability of vaccines in the state at the time of the baseline study.

The training produced changes in all the domains of knowledge, attitudes, and preventive practices of the HCWs. There was a higher level of knowledge among the health workers in all areas; be it in the knowledge of symptoms (above 90%), mode of transmission and preventive measures. Vaccine uptake was observed to have increased within the period although it cannot

be said to be due directly to the intervention. The training was effective in producing the desired change and justifies the use of the theory of change model as a conceptual framework for the intervention. The improvement in the HCWs is in conformity with an intervention reported in Pakistan, where a randomized trial amongst nurses in Pakistan utilized WhatsApp to deliver an educational intervention for 12 weeks reported that the intervention group showed significantly better results in infection prevention control and leadership and communication.[21] Furthermore, the deployment of a COVID-19 training app amongst a group of HCWs in Nigeria led to a significant increase in knowledge from pre-test scores of 54% to 74% at the post-test in conformity with the findings of the present study. These studies[15,21] and ours show the value of the effectiveness of online training in building the capacity of HCWs.

Older HCWs had higher knowledge scores than younger ones in contrast to findings from Ethipia[7] and Nigeria.[15] We posit that the older HCWs were more experienced than their younger colleagues and not just cognitive abilities alone. In addition, it may be that the age group category used in our study (30 years) included many more brilliant but young health care workers. The study did not find any association between knowledge and professional cadre of the HCWs unlike what was found in the UAE[3] and Nigeria[9] and this is probably because of the small sample size in our study. The higher levels of knowledge in Alimosho LGAs and the influence of marital status cannot be easily accounted for. We also did not find any significant association between HCWs' socio-demographic characteristics and preventive practices unlike a study from Ethiopia[7] perhaps on account of the small sample size.

Far more important than the changes observed in the HCWs is the effect of the training on the clients. Contact of the clients with HCWs who had received the intervention led to higher levels of knowledge and better recognition in all domains of knowledge, attitudes and preventive practices examined. There was better recognition of symptoms and of correct preventive practices. Besides, the proportion of those who had received the COVID-19 vaccine rose from less than half to about seven out of ten and were willing to recommend the vaccine to others, although not entirely due to the intervention. The changes confirm the efficacy of the intervention and its benefit not only to the health worker but to their clients. This efficacy of the intervention is like what was seen amongst community members in Iran[17] and Thailand.[22] The finding that single clients had a higher level of knowledge cannot be accounted for. The benefit of clients receiving correct information cannot be overemphasised in the light of conspiracy theories. Contact with health workers who are knowledgeable builds confidence and may lead to sustained utilization of PHCs.

The effectiveness of the intervention is attributable in part to the expertise of the research team, the authentic information in the training materials, the method of delivery, the willingness of the HCWs to participate on account of their perceived benefits and the support given in terms of data provision. Virtual training such as this we undertook has numerous advantages. They are low-cost, non-personal, allow for learning at the individual level and pace, are available for continuous learning. They are invaluable for social distancing and for dealing with large groups. This is probably the way to adopt in this era of the new normal.

Conclusion

This study has shown that virtual training is an effective way to improve the knowledge and skills of health workers who can then impact their clients and bring immense benefit to them, especially in the control of pandemic prone diseases.

Limitations of the study

The positive effect of the intervention amongst HCWs and clients cannot be attributable solely to the intervention as both groups were exposed to other sources of information although not measured which could have contributed to the changes. Mothers were the clients in the study as they were more readily available at PHCs more than men and this limits the generalization of the changes mainly to mothers of children under-five years. The sample size for the study was small. This was in part because the study was meant to demonstrate proof of concept. In addition, the response rate was not as high as expected. However, this is not unusual in studies amongst health workers[5,15] like the participants in this study who for various reasons could not complete the post-intervention assessment.

Recommendations

We recommend the adoption of virtual training to improve the knowledge and skills of HCWs at the first level of care. Such online training should be the new norm and method of continuing professional development. Web-based apps are invaluable, and priority should be given to apps that do not require a lot of data. They should be usable on android phones which are used by more people. Social media for example WhatsApp can be used for educational interventions. Collaboration with the information and technology sector along with telecommunication companies is essential and should be sought.

Funding. The study was funded through a research grant from the Lagos State Research and Innovation Council (LASRIC) won by Professor O.O. Odusanya.

Conflict of Interest. None. The funding agency had no influence in the conduct of the study and the manuscript.

Acknowledgements. We are grateful for the support provided by the Government of Lagos State, the leadership of LASRIC, the leadership of Lagos State University, the Medical Officers of Health of Ikeja and Alimosho LGA, the health care workers and the mothers of under-five children who participated in the study.

Authors' contributions.

OOO- Developed concept, wrote the proposal, developed training modules, participated in the training, supervised data collection and analysis and will serve as guarantor for the study

AA- Participated in proposal writing and training, developed training modules and supervised data collection and analysis

OQB-Participated in training, developed training modules and supervised data collection

BAO- Participated in training, developed training modules and supervised data collection

OE- Developed and managed the training website

OOJ- Supervised data collection

AE- Supervised data collection

All authors were involved in writing the manuscript and approved the final manuscript.

References

1. Centers for Disease Control and Prevention (CDC). "What to do if you are sick". Retrieved 5th May 2020.
2. World Health Organization (WHO). WHO Coronavirus (COVID-19) Dashboard. Available from www.covid19.who.int. Accessed 30 May, 2022
3. Bhagavathula AS, Aldhaleei WA, Rahmani J, Mahabadi MA, Bandari DK. Knowledge and perceptions of COVID-19 among health care workers: cross-sectional Study. JMIR public health and surveillance. 2020; 6: e19160. https://doi.org/10.2196/19160.
4. Al-Ashwal FY, Kubas M, Zawiah M, Bitar AN, Mukred Saeed R, Sulaiman SAS, et al. (2020) Healthcare workers' knowledge, preparedness, counselling practices, and perceived barriers to confront COVID-19: A cross-sectional study from a war-torn country, Yemen. PLoS ONE 15: e0243962. https://doi.org/10.1371/journal.pone.0243962
5. Feldman M, Lacey Krylova V, Farrow P, Donovan L, Zandamela E, Rebelo J, et al. Community health worker knowledge, attitudes, and practices towards COVID-19: Learnings from an online cross-sectional survey using a digital health platform, UpSCALE, in Mozambique. PLoS ONE 2021:16: e0244924. https://doi.org/10.1371/journal. pone.0244924
6. Olum R, Chekwech G, Wekha G, Nassozi DR, Bongomin F. Coronavirus Disease2019: Knowledge, Attitude, and Practices of Health Care Workers at Makerere University Teaching Hospitals, Uganda. Front Public Health. 2020; 8:181. https://doi.org/10.3389/fpubh.2020.00181
7. Yesse M, Muze M, Kedir S, Argaw B, Dengo M, Nesre T, et al. (2021) Assessment of knowledge, attitude and practice toward COVID-19 and associated factors among health care workers in Silte Zone, Southern Ethiopia. PLoS ONE 16: e0257058. https://doi.org/10.1371/journal. pone.0257058
8. Egbi OG, Duru C, Kasia B. Knowledge, attitude and practice towards COVID-19 among workers of a tertiary hospital in Bayelsa State, Nigeria. Pan Afr Med J. 2020;37(Suppl 1):24. doi: 10.11604/pamj.supp.2020.37.24.26259.
9. Tsiga-Ahmed FI, Amole TG, Musa BM, Nalado AM, Agoyi OB, Galadanci HS et al. COVID 19: Evaluating the Knowledge, Attitude and Preventive Practices of Healthcare Workers in Northern Nigeria. Int J MCH AIDS. 2021; 10:88-97. doi: 10.21106/ijma.418.

10. Reuben RC, Danladi MMA, Saleh DA, Ejembi PE. Knowledge, attitudes and Practices Towards COVID-19: An epidemiological survey in North-Central Nigeria. J Community Health. 2020:1–14. https://doi.org/10.1007/s10900-019-00710-0.
11. Odeyemi O, Eyitayo J, Ogunfolaji O, Williams S, Akande M, Akinola O. Knowledge, Attitude, and Practices Towards Preventive Strategies Against COVID-19 Pandemic Among Nigerian Young Adults: A Cross-Sectional Survey. International Journal of Medical Students 2021; 9:257–263. https://doi.org/10.5195/ijms.2021.965
12. Habib MA, Dayyab FM, Iliyasu G, Habib AG. Knowledge, attitude and practice survey of COVID-19 pandemic in Northern Nigeria. PLoS One. 2021;16(1): e0245176. doi: 10.1371/journal.pone.0245176.
13. Chinawa AT, Chinawa JM, Ossai EN, Igwe WC, Nduaguba OC, Aronu AE. Mothers' perception of COVID-19 infection in their under-five children presenting in a tertiary health institution in South-East Nigeria and associated factors. Niger J Med 2021; 30:394-9.
14. Kumbeni MT, Apanga PA, Yeboah EO, Lettor IBK. Knowledge and preventive practices towards COVID-19 among pregnant women seeking antenatal services in Northern Ghana. PLoS ONE 2021;16(6): e0253446. https://doi.org/10.1371/journal.pone.0253446
15. Otu A, Okuzu O, Effa E, Ebenso B, Ameh S, Nihalani N et al. Training health workers at scale in Nigeria to fight COVID-19 using the InStrat COVID-19 tutorial app: an e-health interventional study. Ther Adv Infect Dis. 2021; 8:20499361211040704. doi: 10.1177/20499361211040704.
16. Charan J, Biswas T. How to Calculate Sample Size for Different Study Designs in Medical Research. Indian J Psychol Med 2013; 35(2): 121–126. https://doi:10.4103/0253-7176.116232
17. Honarvar B, Lankarani KB, Kharmandar A, Shaygani F, Zahedroozgar M, Haghighi MRR *et al.* Knowledge, attitudes, risk perceptions, and practices of adults toward COVID-19: a population and field-based study from Iran. *Int J Public Health* **65,** 731–739 (2020). https://doi.org/10.1007/s00038-020-01406-2
18. Paul A, Sikdar D, Hossain MM, Amin MR, Deeba F, Mahanta J, et al. Knowledge, attitudes, risk perceptions and practices of adults toward COVID-19: a population and field-based study from Iran. PLoS One. 2020 Sep 2;15(9): e0238492. doi: 10.1371/journal.pone.0238492.

19. Reinholz DL, Andrews TC. Change theory and theory of change: what's the difference anyway? International Journal of STEM Education 2020 7:2 https://doi.org/10.1186/s40594-020-0202-3
20. Saqlain M, Munir MM, Rehman SU, Gulzar A, Naz S, Ahmed Z, et al. Knowledge, attitude, practice and perceived barriers among healthcare workers regarding COVID-19: a cross-sectional survey from Pakistan. Journal of Hospital Infection. 2020; 105:419–23. https://doi.org/10.1016/j.jhin.2020.05.007 PMID:32437822
21. Jafree SR, Zakar R, Rafiq N, Javed A, Durrani RR, Burhan SK et al. WhatsApp-Delivered Intervention for Continued Learning for Nurses in Pakistan During the COVID-19 Pandemic: Results of a Randomized-Controlled Trial. Front. Public Health 2022; 10: 739761.https://doi: 10.3389/fpubh.2022.739761
22. Maude RR, Jongdeepaisal M, Skuntaniyom S, Muntajit T, Blackseel DS, Khuenpetch W. et al. Improving knowledge, attitudes and practice to prevent COVID-19 transmission in healthcare workers and the public in Thailand. BMC Public Health 2021; 21**:** 749. https://doi.org/10.1186/s12889-021-10768-y

Table 1. Socio-demographic characteristics of PHC workers

Socio-Demographic Characteristics	Pre-training (n=100) N (%)	Post-training (n=63) N (%)
**LGA**		
Ikeja	50(50.0)	39(61.9)
Alimosho	50(50.0)	24(38.1)
**Age-group in years**		
≤30	16 (16. 0)	14 (22.2)
>30	84 (84.0)	49 (77.8)
**Mean (SD)**	40.8±9.6	39.2±9.9
**Gender**		
Female	85(85.0)	54(85.7)
Male	15(15.0)	9(14.3)
**Years of working experience**		
**Mean (SD)**	12.8±9.4	12.5±10.0
≤10	50(50.0)	34(54.0)
>10	50(50.0)	29(46.0)
**Marital Status**		
Married	84(84.0)	47(74.6)
Single	16(16.0)	16(25.4)
**Participated in the IPC training**	70(70.0)	57(90.5)
**Profession**		
**Profession**		
Doctor	16(16.0)	8(12.7)
Nurse	50(50.0)	33(52.4)
CHEW	17(17.0)	11(17.4)
Community Health officer	14(14.0)	9(14.3)
Laboratory scientist	2(2.0)	1(1.6)
Volunteer health worker	1(1.0)	1(1.6)

Table 2. Awareness and knowledge of COVID-19 among PHC workers pre-and post-training

Awareness and knowledge of COVID-19	Pre-test (n=63) N (%)	Post-test (n=63) N (%)
Ever heard of the COVID-19 virus	60(95.2)	63(100)
**Awareness that COVID-19 is real**	60(95.2)	62(98.4)
SARS-COV-2 is the virus that causes Covid-19	52(82.5)	51(81.0)
It's highly infectious	59(93.6)	62(98.4)
The incubation time is 1-14 days	52(82.5)	62(98.4)
The asymptomatic patient can transmit the virus	59(93.6)	62(98.4)
**Knowledge of Symptoms**		
Sore throat	58(92.1)	62(98.4)
Cough	58(92.1)	62(98.4)
Bleeding nose	18(28.5)	14(22.2)
Loss of taste and smell	58(92.1)	61(96.8)
Shortness of breath	59(93.6)	63(100.0)
Fever	58(92.1)	62(98.4)
Joint pain	42(66.7)	48(76.2)
Diarrhoea	36(57.1)	34(54.0)
Rash	17 (27.0)	4(6.3)
Abdominal pain	31(49.2)	27(42.9)
**Knowledge of Mode of Transmission**		
Spread through contact with an infected person	49(77.7)	51(81.0)
Spread through contact with an infected surface	56(88.8)	60(95.2)
Sneezing	59(93.6)	62(98.4)
Talking	49(77.7)	51(81.0)
Spread through coughing	59(93.6)	63(100.0)
**Knowledge of Means of Prevention**		
Hand washing	60(95.2)	63(100.0)
Alcohol-based hand sanitizer	60(95.2)	63(100.0)
Cannot be prevented by alcohol intake	52(82.5)	61(96.8)
Covid-19 vaccine	59(93.6)	61(96.8)
Cough hygiene	58(92.1)	59(93.7)
Social distancing	60(95.2)	61(97.0)
Face mask	60(95.2))	63(100.0)
Covid-19 may lead to serious complication	57 (90.1)	59(93.7)
**Knowledge of Population at Risk of Poor Outcomes from Covid-19**		
Children	35(55.6)	28(44.4)
Pregnant women	49(77.7)	46(73.0)
Elderly	59(93.6)	62(98.4)
People with underlying medical conditions	60(95.2)	63(100.0)
A suspected case can only be ruled after 2 consecutive negative tests	49(77.7)	53(84.1)

Table 3. Pre-and Post-Training knowledge attitude and preventive practices scores of PHC workers

Variable domain	Baseline	Post-training	Statistics	P-value
**Knowledge Score Mean ± SD**	28.48± 2.24	28.52 ± 1.92	0.14**	0.446
**Attitude Score Mean ± SD**	34.44± 3.72	34.83± 3.84	0.63**	0.267
**Practice Score Mean ± SD**	33.95± 3.13	34.60± 2.73	1.81*	0.070

*Wilcoxon signed-rank test **paired t-test

Table 4. Association between PHC workers' socio-demographic characteristics and COVID-19 knowledge scores post-training

Variables	Post Training Knowledge Score Mean ± SD	Test statistics	*P*-value
**Age group (years)**			
≤30	27.57 ± 2.34	-2.17^	0.034*
>30	28.80± 1.71		
**Gender**			
Female	28.52± 1.98	-0.05^	0.479
Male	28.56 ± 1.59		
**Years of Experience**			
≤10	28.44 ± 1.86	-0.37^	0.357
>10	28.62 ± 2.01		
**Marital status**			
Married	28.77 ± 1.56	1.75^	0.043*
Single	27.81± 2.64		
**Participated in any IPC training**			
Yes	28.52 ± 1.96	1.19^	0.425
No	28.67 ± 1.63		
**Received online training on Covid-19**			
Yes	28.47 ± 2.05	0.39^	0.348
No	28.69 ± 1.49		
**LGA**			
Alimosho	29.13±1.70	2.00^	0.025*
Ikeja	28.15± 1.97		
**Level of Education**			
Diploma	28.45 ± 2.42	0.56^^	0.574
Bachelor’s degree	28.44 ± 1.54		
Master’s degree	29.40 ± 2.07		
**Profession**			
Doctor	28.63 ± 2.50	0.27^^	0.847
Nurse	28.55± 1.44		
CHEW, Lab scientist & volunteers	28.15± 2.51		
Community health officer	28.89± 2.20		

^ Independent t-test ^^ One-way ANOVA
*5% significance level

Table 5. Association between the socio-demographic characteristics of the health workers and COVID-19 prevention practice scores

Variables	Post Training Practices Score Mean ± SD	Test statistics	*P*-value
**Age group (years)**			
≤30	34.71 ±3.15	0.00*	1.000
>30	34.57 ± 2.64		
**Gender**			
Female	34.78 ±2.41	0.62*	0.535
Male	33.56 ±4.25		
**Years of Experience**			
≤10	34.71 ±2.86	1.27*	0.203
>10	34.48 ±2.63		
**Marital status**			
Married	34.55±2.68	0.13*	0.899
Single	34.75 ±3.00		
**Participated in any IPC training**			
Yes	34.67 ±2.71	-0.78*	0.436
No	34.00± 3.10		
**Received online training on Covid-19**			
Yes	34.57 ±2.91	-0.73*	0.469
No	34.69 ±2.21		
**LGA**			
Alimosho	35.00±1.74	-0.08*	0.935
Ikeja	34.36±3.19		
**Level of Education**			
Diploma	34.77±2.52	1.33**	0.514
Bachelor's degree	34.33±3.01		
Master's degree	35.80 ±0.45		
**Profession**			
Doctor	34.38±4.21	2.17**	0.539
Nurse	34.39± 2.68		
CHEW, Lab scientists and volunteers	35.23 ±1.96		
Community health officer	34.67±2.65		

*Two-sample Wilcoxon rank-sum (Mann-Whitney) test ** Kruskal-Walli's equality-of-populations rank test

Table 6. Socio-Demographic characteristics of clients

Socio-Demographic Characteristics	Baseline (n=226) N (%)	Post-training (n=133) N (%)
**LGA**		
Ikeja	120(53.1)	75(56.4)
Alimosho	106(46.9)	58(41.4)
**Age-group in years**		
**Mean (SD)**	30.5±5.8	30.9±5.0
≤30	116(51.3)	66(49.6)
>30	121(53.5)	67(50.4)
**Religion**		
Christian	170(75.2)	92(69.2)
Muslim	56(24.8)	41(30.8)
**Level of Education**		
None	1 (0.4)	0(0.0)
Primary	13 (5.8)	4(3.0)
Secondary	112(49.6)	66(49.6)
Tertiary	100(44.3)	63(47.4)
**Marital Status**		
Married	222(98.2)	130(97.7)
Single	4(1.8)	3(2.3)
**Ethnicity**		
Yoruba	116(51.3)	76(57.1)
Igbo	64(28.3)	33(24.8)
Hausa	9(4.0)	2(1.5)
Others	37(16.4)	22(16.5)
**Average Monthly Income**		
**Mean (SD)**	49,265.6±8,6673.2	
≤ ₦50,000	177(78.3)	103(77.4)
>₦50,000	44(19.5)	28(21.1)
Non -Response	5(2.2)	2(1.5)
**Employment Status**		
Employed	186(82.3)	117(87.9)
Unemployed	40(17.7)	16 (12.0)

Table 7. Client's awareness and knowledge of Covid-19 before and after interaction with trained PHC workers

Awareness and knowledge of covid-19	Before (n=133) N (%)	After (n=133) N (%)
Ever heard of the Covid-19 virus	131 (98.5)	132(99.2)
**Source of Information**	**(n=131)**	**(n=132)**
Media	125(95.4)	130(98.5)
Religious centres	58(44.3)	128(97.0)
Official website	31(23.7)	75(56.8)
**Awareness that Covid-19 is real**	106(80.9)	131(99.2)
It's highly infectious	105(80.2)	127(96.2)
An asymptomatic patient can transmit the virus	95(72.5)	120(90.9)
**Knowledge of Symptoms**		
Sore throat	108(82.4)	124(93.9)
Cough	122(93.1)	130(98.5)
Bleeding nose	73(55.7)	73(55.3)
Loss of taste	90(68.7)	114(86.4)
Shortness of breath	112 (85.5)	123(93.2)
Loss of smell	83(63.4)	112(84.8)
Fever	114(87.0)	129(97.7)
Joint pain	46 (35.1)	74(56.1)
Diarrhoea	54(41.2)	96(72.7)
Rash	39(29.8)	26(19.7)
Abdominal pain	48(36.6)	52 (39.4)
**Knowledge of Mode of Transmission**		
Spread through contact with an infected person	117(89.3)	128(97.0)
Spread through contact with an infected surface	122(93.1)	126(95.5)
Sneezing	126(96.2)	131(99.2)
Talking	110(84.0)	110(83.3)
Spread through coughing	125(95.4)	132(100.0)
**Knowledge of Means of Prevention**		
Staying at home	124 (94.7)	122(92.4)
Hand washing	129(98.5)	131(99.2)
Alcohol-based hand sanitizer	129(98.5)	130(98.5)
Cannot be prevented by alcohol intake	48(36.6)	98(74.2)
Covid-19 vaccine	85(64.9)	123(93.2)
Cough hygiene	129(98.5)	130(98.5)
Social distancing	129(98.5)	132(100.0)
Face mask	130(99.2)	132(100.0)
Covid-19 may lead to serious complication	116 (88.5)	126(95.5)
Doctor/nurse talked about Covid-19 outbreak	95(72.5)	130(98.5)

Table 8. Knowledge, attitude, and preventive practices of clients before and after interaction with trained PHC workers

Mean ± SD	Baseline	Post-training	Test statistics	*P*-value
**Knowledge Score**	20.00± 4.25	22.96 ± 2.32	-7.03^	<0.001
**Attitude Score**	26.50 ± 4.01	28.74 ± 5.20	4.63^^	<0.001
**Practice Score**	26.39 ± 5.28	28.47± 4.61	3.85^^	<0.001

^Wilcoxon signed-rank test
^^paired t test

Table 9. Association between the socio-demographic characteristics of clients and knowledge scores after interaction with trained PHC workers.

Sociodemographic characteristics	After intervention (Mean ± SD)	Test Statistics	*P*-value
**Age group (years)**			
≤30	23.08± 2.52	-0.92^	0.359
>30	22.85± 2.11		
**Religion**			
Christian	23.21 ± 2.19	1.70^	0.090
Muslim	22.41 ± 2.54		
**Marital status**			
Married	22.91 ± 2.31	-2.07^	0.038*
Single	25.33 ± 1.15		
**Ethnicity**			
Yoruba	22.62 ± 2.48	3.73^^	0.292
Igbo	23.39± 2.24		
Hausa	22.50 ± 0.71		
Others	23.55 ± 1.77		
**Monthly Income**			
≤₦50,000	22.89 ± 2.27	1.23^	0.218
>₦50,000	23.32 ± 2.54		
**Employment Status**			
Employed	22.97 ± 2.35	0.28^	0.779
Unemployed	22.88 ± 2.16		
**LGA**			
Alimosho	22.31± 2.71	-2.45^	0.014*
Ikeja	23.47 ± 1.83		
**Level of education**			
Primary	21.75 ± 2.06	2.05	0.360
Secondary	23.09 ± 2.38		
Tertiary	22.90 ± 2.28		

^ Two-sample Wilcoxon rank-sum (Mann-Whitney) test

^^ Kruskal-Walli's equality-of-populations rank test

* 5% significance interval

Table 10. Association between the socio-demographic characteristics of clients and preventive practice scores after interaction with trained PHC workers

Variables	Post-test score (Mean ± SD)	Test statistics	*P*-value
**Age group (years)**			
≤30	27.92 ± 4.06	1.37^	0.087
>30	29.01 ± 5.07		
**Religion**			
Christian	28.09 ± 4.61	1.45^	0.074
Muslim	29.34 ± 4.54		
**Marital status**			
Married	28.38 ± 4.56	1.60^	0.056
Single	32.67 ± 5.77		
**Ethnicity**			
Yoruba	28.68 ± 4.61	1.23^^	0.302
Igbo	28.91 ± 4.88		
Hausa	23.50 ± 3.54		
Others	27.54 ± 4.15		
**Monthly Income**			
≤₦50,000	28.33 ± 4.48	0.67^	0.507
>₦50,000	29.00 ± 5.28		
**Employment Status**			
Employed	28.43 ± 4.59	0.31^	0.755
Unemployed	28.81 ± 4.92		
**LGA**			
Alimosho	29.59 ± 4.57	2.49^	0.014*
Ikeja	27.61 ± 4.48		
**Level of education**			
Primary	27.00 ± 4.08	1.79^^	0.170
Secondary	27.82 ± 4.75		
Tertiary	29.25 ± 4.42		

^ Independent t-test * 5% significance interval
^^ One-way ANOVA F test value
