# Supplementary material for: Building capacity of primary health care workers and clients on COVID-19: Results from a web-based training
Source: PLoS One. 2022 Oct 7;17(10):e0274750. doi: 10.1371/journal.pone.0274750 (PMC9543989; doi:10.1371/journal.pone.0274750)
Supplement: Attachment — Submitted filename: Response to Reviewers_August29_2022.docx [file pone.0274750.s003.docx]

A. **Response to Journal requirements**
1. Please ensure that your manuscript meets PLOS ONE's style requirements, including those for file naming.

**Response.** This has been done. The two files are labelled appropriately as well as the response to reviewers

2. PLOS ONE does not copy edit accepted manuscripts

**Response**. This has been done. The submission is free of typos and grammatical errors.

3. Please include your full ethics statement in the ‘Methods’ section of your manuscript file. In your statement, please include the full name of the IRB or ethics committee who approved or waived your study, as well as whether or not you obtained informed written or verbal consent. If consent was waived for your study, please include this information in your statement as well

**Response**. Ethical approval was obtained from the Health Research Ethics Committee of the Lagos State University Teaching Hospital (REFERENCE NO: LREC/06/10/1501). Written informed consent was obtained from all the participants after explaining the study's aim and objectives. (This was in the initial submission, lines 258-260)

4. In your Data Availability statement, you have not specified where the minimal data set underlying the results described in your manuscript can be found. PLOS defines a study's minimal data set as the underlying data used to reach the conclusions drawn in the manuscript and any additional data required to replicate the reported study findings in their entirety. All PLOS journals require that the minimal data set be made fully available. For more information about our data policy, please see <http://journals.plos.org/plosone/s/data-availability>.

Upon re-submitting your revised manuscript, please upload your study’s minimal underlying data set as either Supporting Information files or to a stable, public repository and include the relevant URLs, DOIs, or accession numbers within your revised cover letter. For a list of acceptable repositories, please see <http://journals.plos.org/plosone/s/data-availability#loc-recommended-repositories>

**Response**. Data set is now uploaded as an Excel sheet

5. Please review your reference list to ensure that it is complete and correct. If you have cited papers that have been retracted, please include the rationale for doing so in the manuscript text, or remove these references and replace them with relevant current references. Any changes to the reference list should be mentioned in the rebuttal letter that accompanies your revised manuscript. If you need to cite a retracted article, indicate the article’s retracted status in the References list and also include a citation and full reference for the retraction notice.

**Response**. Two new references have been added (no 16 and 21) making 24.

B. **Responses to Reviewers (Line numbers in red are as on the tracked changes document)**
1. The statistical analysis in general is appropriate and rigorous. However one is curious to know why an "independent t-test" was used to measure differences in knowledge, attitude and practice (KAP) pre- and post- intervention instead of a "paired t-test"?

**Response**. **Line 269-272**.The independent T test was used when we compared post training scores only for analysis of statistical association (Tables 4 and 5) but when we compared as on Table 3, the paired T test was used. This we believe is appropriate.

“Associations between the socio-demographic characteristics of respondents and their post-intervention COVID-19 knowledge, attitude, and preventive practices were assessed using an independent sample t-test and one-way analysis of variance (ANOVA) when data were normally distributed or Wilcoxon signed-rank and Kruskal-Wallis equality-of-populations rank test as appropriate when the data were not normally distributed” This was as in the first submission.
2. Line 89 – 90: Almost 80% had positive - had positive what?
82% used face masks - desirable to know what use of face mask means in the study. Is it consistent use of face mask or ever use of face mask?

**Response**. **Line 86**. Positive attitudes (80%)

**Line 87. Face masks use was consistent**
3. Line 127 – 128: The population is estimated to be over 20 million.- Provide reference

**Response. Line 124. Reference 16 now included.**
4. The health care of its residents is overseen by the Lagos State Ministry of Health. - It is also true that the Federal Ministry of Health also play a role in the health of the residents. Please clarify what is meant by 'overseen'

**Response.** The Statement has now been re-written

**Line 125-127 “**Health services for the citizens are available through both public and private facilities. The larger numbers of publicly owned facilities in Lagos State belong to the Lagos State Government although there are a few owned by the Federal Government”
5. Line 134 – 135: Government-owned health facilities include 18 PHC centres and the Lagos State University Teaching Hospital (LASUTH). - Is there no secondary health facility in the LGA? Mention the presence or absence of secondary health facility in the LGA.

**Response.** **Line 135-136**. There is no secondary health care facility in Ikeja LGA as the existing one was upgraded to become the teaching hospital (LASUTH).
6. Line 158 – 159: PHCs were selected based on having a high patient load and all eligible health workers in the selected facilities were recruited into the study - How many PHCs were selected in each LGA. The sampling method for selecting those PHCs not mentioned

**Response.** **Line 163-165**. PHCs were selected based on having a high patient load (four facilities in Ikeja LGA and five facilities in Alimosho LGA) and all eligible health workers in the selected facilities were recruited into the study. These PHC were selected by simple random sampling.
7. Line 191: …. their first visit ….. - Does this mean their first ever visit/contact to facility or first visit for that episode of illness. Please clarify

**Response. Line 201** First visit for childcare during the study
8. Line 263 – 264: The mean ages at the baseline and the end of the study were 40.8±9.6 and 39.2±9.9 years respectively. - What is the explanation for the change in age at baseline and at the end of the study? If it is the 63 that completed the study that were used then their ages shouldn't have changed remarkably. Indicate the duration of each phase of the study in the methods section.

**Response**. **Line 281**. The observation by the reviewers was correct but we have now effected the appropriate changes. Only the 63 that completed had their menage computed. The baseline mean age initially used was for all the recruited participants, but this has now been removed see Table 1

**Line 203,214,229-231.The duration of the phase of the study:** baseline -four weeks; intervention four weeks. Post intervention data collection.one week. Now indicated in the methods section
9. Line 270 – 271: …… level of knowledge was 270 high on symptoms, modes of transmission, and preventive measures - Provide the figure for the level of knowledge. It is not enough to state that it is high except in the discussion section.

**Response**. **Line 290-291**.The details are now provided

The level of knowledge was high on symptoms (< 90%), modes of transmission (<80%), and preventive measures (<93%).
10. Line 278 – 279: Marital status and LGA of health workers were statistically significantly associated with knowledge scores - In what direction are the differences?

**Response**. **Line 298-300**. The direction has now been shown

Married PHC workers had higher mean knowledge scores compared with the single. PHC workers in Alimosho LGA had higher mean knowledge scores than those in Ikeja LGA.
11. Line 290: …….. interactions with trained PHC workers … - Provide information on the nature of their interactions (duration of interactions, mode of interaction - one - on - one or group health talk, etc) with PHC workers in the methods section.

**Response**. **Line 230-233**. This has been done in the methods section

After the training, the same set of clients were exposed to the trained PHC workers for one-on one counselling on invitation to the PHC. Each session lasted for about ten minutes.

12. Line 298 – 299: The interaction with trained PHC workers led to an increase in all aspects of the knowledge enquired about: - Provide data showing the increase as provided for the baseline.

**Response.** **Line 321-329**. The data was provided as before on Table 7

The interaction with trained PHC workers led to an increase in clients’ knowledge of symptoms (<80%), modes of transmission (<80%) and preventive measures (<90%).

Single clients had significantly higher mean knowledge scores than the married but clients in Ikeja LGA had significantly higher knowledge scores than those in Alimosho LGA
13. Line 326 - ….. cough. - delete - cough is repeated in this sentence
**Response**. **Line 336**. The repeated word “Cough” is now deleted.
14. Study limitation - It is desirable to mention the limitation in the study design - The lack of control group

**Response.** Line 439-440. Now done

The lack of a control group is another limitation of the study.

**Professor Olumuyiwa Odusanya (signed)**

**Lead and Corresponding Author**
